# The critical role of psychosomatics in promoting a new perspective upon health and disease

**Published:** 2009-11-25

**Authors:** D Dragoş, MD Tănăsescu

**Affiliations:** * ‘Carol Davila’ University of Medicine Bucharest, 1^th ^Internal Medicine Department, University Emergency Hospital BucharestRomania

**Keywords:** somatoform disorders, unexplained physical symptoms, somatization, amplification, alexithymia, cognitive model, cytokine

## Abstract

In an evolutionary model, health and disease are regarded as successful and respectively failed adaptation to the demands of the environment. The social factors are critical for a successful adaptation, while emotions are means of both signaling the organism's state and of adapting the physiological responses to environmental challenges. Hence the importance of a biopsychosocial model of health and disease. Psychoemotional distress generates and/or amplifies somatic symptoms. Somatization may be viewed as an altered cognitive process, inclining the individual to an augmented perception of bodily sensations and to an increased degree of complexity in reporting negative experiences (hence the greater cognitive effort allocated thereto). Somatosensory amplification and alexithymia are key elements in this process. The brain's right hemisphere is more involved in the generation of emotionally conditioned somatization symptoms. Somatic symptoms have various psychological and social functions and are strongly influenced by the particular belief system of the individual. Inappropriately perceiving the environment as an aggressor and excessively responding to it (by activating the cytokine system in correlation with the arousal of the psychic, nervous, and endocrine systems) may be a key element in the altered cognition conducive to ill health.

## Introduction

The conventional medicine has no comprehensive theory about the meaning of some fundamental concepts, such as health, predisposition to disease (to ill health) and disease. One cannot miss the correlation with an old dilemma of both philosophy and science: is the dichotomy mind–body correct or should those two be regarded as forming an inseparable unity. The current conventional explanations regarding the concept of illness are based on the pathological findings brought on by infection, substance poisoning, trauma and genetic mutation. The reason is the influence exerted by the precepts of classical physics upon the medical concepts, to which they have imparted a mechanistic, materialistic, deterministic, reductionist orientation, characterized by a linear cause–effect type of vision and a strong predilection to handy explanations about disease. Many attempts have been made lately to build a more comprehensive theory, able to integrate the present proven roles of physical, social, environmental, and psychological factors in disease predisposition, etiology, and pathogenesis. The psychic background might be viewed as either cause or effect and as either aggravating or accompanying factor of symptoms or physical illnesses.

## Evolutionary models

Weiner talks about the necessity of an evolutionary vision, able to lead to a unified, integrated theory about health and disease and to a more clear taxonomy in medicine, taking as a start point Darwin's theory, which asserts that evolution, is spurred by natural and sexual selection. Natural selection acts differently upon individuals according to their ability to adapt: those able to adapt survival to reproduce; failing to adapt reduces the aptitude to reproduce and to be successful, finally leading to disease or death. This formulation may be extrapolated to (the predisposition to) disease, which may be viewed as failed adaptation, whereas health would equate with successful adaptation. 

Adaptation is determined by multiple genetic, morphological, physiological and behavioral factors and allows the organism to accommodate with the multiple and varied selective pressures. There is evidence that primates with a social life (i.e. living in groups) have a selective advantage and are among the species with the greatest evolutionary success. The social behavior (for instance, the social support) seems to increase the individual's chances to survive and to be fit for reproduction. The physiological (immunological, metabolic, cardiovascular) and behavioral adaptations are specifically directed by interactions with the environment, while emotions have emerged on the evolutionary line as ways of signaling the state of the organism and of adapting the physiological responses to environmental challenges [1]. 

## Biopsychosocial models

Efforts made for many decades regarding the aim of finding a unified concept of health and disease have been channeled toward developing a new medical model able to integrate psychic and social aspects in a biopsychosocial model (proposed originally by Engel). According to this model, comprehending pathological processes should rely upon understanding the interaction of three entities: the body (bio–), the mind (psycho–) and the social context. Therefore, the disease process should be approached from the perspective of a complex multifactorial model [2]. Not only a new model of disease is necessary, but also a new diagnosing system relying on the psychosomatic model, instead of the biomedical model of disease (upon which the diagnosing system currently used in the conventional medicine is based). Oken describes a multiaxial method, which allows a diagnosis formulation reflecting the adaptation process, including the biological, psychological and social factors. This approach may give rise to a momentous reform in medical care, reflecting the psychosomatic model of disease [3].

The original trend in psychosomatic medicine was to correlate the physical symptoms only with individual psychic aspects, disregarding the social environment the individual is living in. This approach is nowadays anachronic, and the resurrection of psychosomatic medicine depends precisely on this comprehensive vision upon the individual as part of the environment [4]– integrating the relationship between personality and the social matrix of somatic distress is critical in understanding the somatoform disorders [5].

Somatization is a universal phenomenon, identifiable in any culture or civilization [6]. There are significant differences in the somatization process, which are not leveled out by a health care system equally accessible to any ethnic group. Kirmayer and Young have proved that the somatic symptoms have various psychological and social functions and are strongly influenced by the particular system belief of the individual. Depending on the cultural, ethnic, religious, social, professional, and familial environment of the individual, the symptoms may be regarded as signifying:

- a physical disease;
- a functional disorder;
- a psychic illness;
- the outward expression of inner psychic tensions;
- a culturally coded expression of distress;
- a mean of channeling social dissatisfaction;
- a way o defining or redefining the individual's position in relation to other people.

Different ethnic groups have various cultural/historical/ religious backgrounds, which are reflected in the:

- different ways of perceiving and expressing distress, including the bodily one;
- different sets of ethnomedical beliefs;
- different perceptions about the official health care system, its usefulness and its accessibility.

Therefore, the vision about somatization should be broadened so as to take into account not only the individual characteristics, but also the social and cultural ones [7]. Thus we will be able to surpass the nosological limitations imposed by the current psychiatric theories [8].

## Psychophysiological models 

Two terms are used in literature to designate somatization phenomena: somatoform disorders (SFD) and medically unexplained physical symptoms (MUPS). The general trend is to consider the two terms as completely interchangeable.

Rief et al. found out that the most common somatoform symptoms are pain (primarily, back pain, joint pain, pain in extremities, and headache), abdominal symptoms (bloating or intolerance to several foods) and cardiovascular symptoms (palpitations) [10].

It is generally recognized the propensity of people with intense and/or persistent feelings to push the emotional distress towards a physical territory. Therefore, somatic distress may be viewed as a distinct psychological dimension [9]. Psycho behavioral characteristics have an important predictive value for SFD and should probably be used as positive diagnostic criteria for these disorders [11].

While exploring the psychology of physical symptoms, researchers have looked for various patterns of somatic displacement of trauma, frustrations and resentments. Psychophysiological models, based on the notion of combined effects (the psychic and physiological levels regarded as being in an indissociable interplay) [12] are increasingly accepted more at present. New paradigms are also necessary for defining the role of psychotherapy for the subjects with complex disorders and chronic symptoms [13].

Psychoemotional distress generates and/or amplifies somatic symptoms, but many other variables intervene in modulating their intensity and the manner they are perceived, interpreted, and reported by the patients – gender, race, and ethnic and cultural background are only a few among them [14, 15, 16]. The result is the multifaceted polymorphism of somatization, although its mechanism seems to be in essence only one. This made some authors hypothesize that the apparently manifold somatoform syndromes might be considered as being one and the same under a variety of appearances [17]. 

Gender seems to have an important bearing upon symptom perception. Kroenke and Spitzer have shown that most physical symptoms (either explained or unexplained by physical causes) are characteristically reported at least 50% more frequently by women than by men. The manner of reporting the symptoms is influenced most strongly by the concurrent depressive and/or anxious disorders (which are also more prevalent in women). However, the demographic factors have an independent effect too. Among these, gender is the most important, followed by the educational level. Age and race have a lesser effect, and so do medical comorbidities [18].

The severity of somatization is related to the personality traits and the psychiatric disorders [19]. Some of these originate in childhood: learning to adopt the sick role and being encouraged to do so in the first decade of life predicts the illness behavior during adulthood [20]. In fact, some researchers consider the somatoform disorders as being severe psychiatric diseases unduly disregarded by the psychiatrists, given their high prevalence in the general population [21].

## Cognitive models

The current tendency, born decades ago, is to perceive somatization as an altered cognitive process, which is the result of the interaction between the cognitive characteristics and the social ones [22]. The alteration of the cognitive process in patients with SFD can be demonstrated objectively by means of laboratory studies (for example, by using the technique of evoked response potentials, either auditory [23] or visual [24]). The somatization process may have its roots in or may be amplified by an altered perception of disease–therefore understanding this process requires an assessment of the cognitive representation of illness [25]. The alteration of the cognitive skills is reflected in the amplification of bodily sensations, which fuel the tendency to somatization and therefore the proneness to develop SFD [26]–hence, the recognized effectiveness of cognitive–behavioral therapies in the management of SFD [27], documented by many controlled clinical trials [28]. Interpreted from a cognitive–psychobiological perspective, SFD may be explained through the readiness of somatization– prone persons to notice and remember physical symptoms, which might indicate certain psychological and psychophysiological mechanisms possibly involved in maintaining the SFD. It was shown (Rief et al.) that the patients with SFD (if compared with normal subjects) have a higher level of salivary cortisol, an increased cardiac frequency, and a lower digital pulse volume while executing a stress–generating task; they also tend to report a higher degree of psychic distress [29].

The alteration of the cognitive processes is involved not only in the genesis of SFD, but also in the health state deterioration. Thus, health outcome is affected by the cognitive aspects generated by the negative emotional impact of recalling some unhappy events. This is related to the increased complexity of the cortical processes involved in the integration of the episodes in question, reflecting the amount of intellectual resources allocated to the management of those conditions or problems. Recalling negative experiences requires an increased cognitive effort, reminiscent of that induced by the original confrontation with the negative experiences. The cognitive involvement, both excessive and deficient, is associated with a diminished well–being sensation as compared with a moderate cognitive involvement. This has implications upon the manner in which the negative events are reconstituted in memory, upon the relationship between the cognitive processes and the emotional ones, as well as upon the relationship between cognitive processes and health state. In an effort to unravel whether very unpleasant life events are recalled at a different level of complexity than the neutral memories and whether the differences in complexity are correlated with the health outcome, Suedfeld and Pennebaker have asked volunteers to give written accounts about both negative life experiences, and trivial events. The investigators have compared the complexity scores of these two types of reports and have correlated them with an assessment of the well–being state. The reports about negative experiences have been significantly more complex, which implies greater cognitive effort allocated thereto. In the subjects who wrote about negative events, a significant relationship between the report's complexity and the general state improvement was apparent [30]. Solano et al have reached similar results [31].

Lane points out the importance of emotional awareness, i.e. the subject's ability to have a conscious grasp on his feelings and to be able to express them. Passing from the unconscious to the conscious level is seen as a gradual continuous process of acquiring an increasingly refined cognitive skill with potentially positive health–preserving effects [32].

The intricate relationship between psychic and physical levels in SFD is emphasized by the identification of a predominant pattern of lateralization of somatic symptoms correlated with emotional disorders. Min and Lee have studied patients with depressive, anxiety and somatization disorders (divided into two groups according to the laterality of somatic symptoms: either left or right) and found that the main somatic symptoms (especially headache, but also elsewhere localized pain) occur more frequently on the left side, with no significant differences among the left–and right– sided groups as to demographic variables (such as age, gender, marital status, educational level), diagnosis, and duration of illness. Moreover, the anxiety and depression scores were higher in the left–sided group, without attaining statistical significance. The authors infer that the brain's right hemisphere has more to do (than the left one) with the occurrence of emotionally conditioned somatization symptoms [33].

One cannot draw a neat boundary between somatoform symptoms and the so–called medically unexplained physical symptoms (MUPS). The MUPS may be considered as a variant of the somatoform disorders, from which they differ by a more diffuse, less differentiated nature. Hence the large overlap among the various syndromes classified as MUPS [34]. Some authors defined them as medical symptoms with no identifiable pathology and pointed out their relationship with psychiatric disorders, childhood and adulthood trauma, and personality traits [35], arguing that a paradigm change is mandatory in our approach to MUPS [36], all the more so as they are highly prevalent both in general population, and among individuals that frequently attend health care facilities [37]. The failure to provide medical explanations for such symptoms joined with the patients' disturbed psychic background (which is intimately involved in the disease outcome) is conducive to the heavy demands put upon the health care system [38, 39]. An excessive amount of money is spent for the management of patients complaining of multiple MUPS (‘polysymptomatic somatizers’), in whom the standard medical treatment is doomed to failure from the start and in whom the psychosocial interventions, although apparently beneficial, have not been proven to have a lasting and clinically significant impact [40]. 

One problem is the patients' refusal to admit psychosomatic explanations for their symptoms, understandable as many of them do not perceive a sufficiently strong correlation between their symptoms and their psychological states [41].

It is surmised that somatosensory amplification and alexithymia play an important predisposing role in the pathogenesis of MUPS. The somatosensory amplification is the tendency to report somatic sensations as being intense and worrisome, while alexithymia is a personality feature characterized by the subject's difficulty to recognize his own emotions, paralleled by a tendency to focus upon external events and upon bodily sensations–the two phenomena are frequently correlated, both having an important role in psychosomatic patients [42]. The alexithymic individual may not be able to recognize the psychic impact of the stress and/or psychic distress generating circumstances [43]. He/she tends to displace his attention from those circumstances to the various somatic symptoms occurring in the context (with an ensuing increased tendency to be anxious and to report physical symptoms [44]). The result is his/her inclination to minimize the importance of therapeutically approaching the emotional disturbances. In such cases, focusing strictly upon the physical manifestations without granting due importance to the psychic trauma may render unfruitful the cure–aimed attempts [45]. Alexithymia has a recognized role in both medical and psychiatric disorders [46], some authorities considering it as a key element in a correct vision about the psychosomatic process, with a proven predictive value in somatizing patients [47], clearly related to the demands put upon the health care system [48] and to health behavior [49]. Although considered to be involved mainly in the genesis of SFD [50], alexithymia seems to play a role also in the psychogenesis of physical illnesses [51]. However, some studies provide only modest evidence for the role of alexithymia in predicting the advent of somatic symptoms in medically unexplained illnesses (such as chronic fatigue syndrome [52]) and or in predicting their persistence [53].

Nevertheless, there are opinions that MUPS cannot be considered exclusively psychic disorders, if one takes into account the fact that the most common somatic functional syndromes (among these: functional dyspepsia, irritable bowel syndrome, fibromyalgia, chronic fatigue syndrome, all of which might be put into the MUPS category), despite being related to depression and anxiety, are not entirely dependent on either of these [54].

The psychosomatic medicine suggests that the concept of distress/disorder/disease should include, generically and comprehensively, the whole range of disagreeable sensations/conditions experienced by the human being, from those on the mental–emotional level, to those on the physical one, including both functional symptoms (in which the disturbance affects only the physiology of the system, with no structural changes), and organic diseases (in which morphological alterations are apparent). In fact, the concept of disease should be envisaged as a continuum, if we take into account:

- the different intermediate or borderline variants;
- the variable severity of organic lesions;
- the definition of nosological entities by alterations whose identification implies sophisticated tests, available only in specialized centers.

The human being should be regarded as being in a continuous interaction with his/her environment, which might be viewed as a potential aggressor. For thousands of years until the middle of the last century, the environment's assault had been predominantly on the physical level, with trauma and infectious diseases being the main cause of death. Only by the middle of the last century, the level of aggression shifted toward the psychoemotional level, as human society had become increasingly more sophisticated, imposing ever–higher standards to the individual (that is what we generically call psychosocial stress). Needless to say, this trend has been continuing since then

The response of the organism confronted with these (physical, chemical, biological, psychoemotional, social) assaults is to defend itself by putting into action, as a nonspecific mean, the inflammatory system, and, as a specific mean, the immune system. Actually, the immune system may be regarded as an evolutionary requirement, since the reactions generated by the inflammatory system may endanger not only the aggressor's integrity, but also that of the host organism, which it is actually supposed to protect (the actions of the two systems cannot be in fact dissociated). In the orchestration of these defense responses come into play the main coordination systems of the organism: the psychic, the nervous (primarily the autonomic one), and the endocrine. But the real switch of this response (the element which makes the link between the regulatory systems on one hand and the inflammatory and immune systems on the other hand) seem to be the cytokines

The nervous and endocrine systems have been for a long time regarded as an organic unity. Several neuroendocrine axes have been identified: hypothalamus–pituitary–adrenal (HPA) axis, hypothalamus–adrenal medulla (HAM) axis, hypothalamus–pituitary–gonadal (HPG) axis. Among these, the HPA axis and the HAM axis have a decisive role in the organism's response to stress – both affect and are affected by the production of cytokines: IL–1 and IL–6 stimulate the release of corticotropin– releasing hormone (CRH) from the hypothalamus via the eicosanoids pathway mediated by the cyclooxygenase [55], while the diurnal rhythm and exercise–induced changes in the plasma cortisol level differentially regulate the production of IL–1beta, IL–6 and tumor necrosis factor–alpha (TNF–alpha): it has been shown that IL–6 production is highly sensitive to these changes, while the TNF–alpha production is comparatively resistant [56]. 

The psychological modulation of the immune function through psychosocial stressful factors or through psychosocial therapeutic interventions may alter the health state [57]. The cytokines are an essential element in the bidirectional communication between the immune system and the brain. Understanding their role provides a critical insight in the influence behavior, mood and cognitive function exert upon the immune system [58].Several mechanisms of the cytokines–brain interaction have been pointed out, relevant both for internal diseases, and for neurologic and psychiatric disorders [59, 60, 61]. Moreover, the level of interleukins may be altered by affective, cognitive [62], behavioral factors, with implications in understanding the behavior and the immunopathology [63]. 

It is already known that the proinflammatory cytokines can influence the onset and the outcome of an entire spectrum of diseases (not only the cardiovascular ones) and that their action may have debilitating and disabling consequences. On the other hand, the output of proinflammatory cytokines may be directly stimulated by negative emotions and by stressful experiences (not only by chronic or relapsing infections). Accordingly, the disturbance of the immune regulation induced by emotional distress might be a key mechanism whereby the negative emotions jeopardize health. Therefore, psychoneuroimmunology has a broad range of implications for the fundamental biological sciences and for medicine. 

Revisiting the concept of the environment as an aggressor, one might consider it as appropriate when the individual is threatened by mechanical, physical, chemical, biological, and psychological potentially noxious agents/factors, but as inappropriate when neither of these is present and/or the individual is overreacting. This lack of adequacy may be envisaged as expression of a disturbed cognitive process, leading to an altered perception of the environment, and therefore to failed adaptation.

## Conclusions

models of health and disease (evolutionary, biopsychosocial, psychophysiological, cognitive) have been elaborated in order to account for the role of psychosocial factors in the genesis of ill health. Each has its merits and its drawbacks, and a critical analysis may perceive all of them as part of a single, unifying vision. Somatization may be regarded as an altered cognitive process, inclining the individual to an amplified perception of the bodily sensations and of the negative experiences. Inappropriately perceiving the environment as an aggressor and excessively responding to it (by activating the cytokine system in correlation with the arousal of the psychic, nervous, and endocrine systems) may be a key element in the altered cognition conducive to ill health.
